# Tree-ring width and δ^18^O-derived hydroclimatic reconstructions allow a distinction between soil and atmospheric drought in the Mountain Forests of Northeastern Iran

**DOI:** 10.1038/s41598-026-52364-3

**Published:** 2026-05-19

**Authors:** Zeynab Parisa Foroozan, Mohammad Hossein Mazaherifar, Sugam Aryal, Jussi Grießinger, Kambiz Pourtahmasi, Achim Bräuning

**Affiliations:** 1 Institute of Geography, Friedrich-Alexander-Universität Erlangen-Nürnberg (FAU), 91058 Erlangen, Germany; 2 Department of Environment and Biodiversity, Paris-Lodron-Universität Salzburg, 5020 Salzburg, Austria; 3 Department of Wood and Paper Science & Technology, Faculty of Natural Resources, University of Tehran, Karaj, 31587-77871 Iran

**Keywords:** Multi-proxy reconstruction, Drought reconstruction, Altitude correction, Semi-arid mountain forests, Forest drought resilience, Climate sciences, Environmental sciences, Hydrology, Natural hazards, Water resources

## Abstract

Iran’s long history of climate-related crises, primarily driven by droughts, has been intensified by ongoing climate change, placing forest ecosystems under increasing hydroclimatic stress. In recent decades, prolonged droughts combined with elevated atmospheric moisture deficits have reduced ecosystem resilience and increased vulnerability to degradation. To better understand long-term drought dynamics and their ecological impacts, we developed two 200-year chronologies (1821–2020) of tree-ring width (TRW) and stable oxygen isotope variations (δ^1^⁸O) from *Juniperus polycarpos* in the Hezar Masjed Mountains, northeastern Iran. The δ^1^⁸O record served as a proxy for atmospheric moisture conditions and was used to reconstruct growing-season (March–September) vapor pressure deficit (VPD). When combined with TRW in a multiple regression framework, this dual-parameter approach enabled reconstruction of the Standardized Precipitation-Evapotranspiration Index (SPEI07), representing cumulative growing-season hydroclimatic conditions related to soil moisture availability. This allows the differentiation of atmospheric and soil drought impacts on tree growth over two centuries. By classifying drought years into VPD-only, SPEI-only, and combined drought events, we found that drought conditions associated with reduced soil moisture availability (SPEI) exerted the strongest constraint on radial growth. Tree growth declined most strongly during severe SPEI droughts, followed by severe combined drought (COMB-D) years, whereas atmospheric drought alone (VPD-D) had a weaker and more transient effect. Growth typically recovered within two years following drought events. Analysis of long-term drought classifications (1821–2020) revealed a shift towards more intense droughts in recent decades, particularly in the frequency of severe VPD and combined drought years. Our findings highlight that tree growth in semi-arid mountain ecosystems is primarily limited by soil moisture availability, with atmospheric drought acting as an additional stressor when coinciding with soil moisture deficits. This study demonstrates the value of combining multiple tree-ring proxies to disentangle drought mechanisms and improve understanding of forest responses to climate change.

**Supplementary Information:**

The online version contains supplementary material available at 10.1038/s41598-026-52364-3.

## Introduction

Understanding how ecosystems respond to long-term climate variability is crucial, particularly in regions increasingly affected by drought and water scarcity. Iran, dominated by arid and semi-arid climates typical of large areas of southwest Asia, is highly vulnerable to climate change and its compounding impacts on ecosystems and human societies^1,2^. Nearly 65% of Iran’s land area is classified as arid and further 25% as semi-arid, underscoring the country’s general high susceptibility to warming and drying trends^3,4^. In recent decades, climate change has exacerbated Iran’s already serious hydrological challenges, driving higher temperatures, altered precipitation regimes, and prolonged drought episodes. Meteorological observations recorded widespread increases in temperature extremes, including more frequent heat waves and warm nights and days, coupled with a decline in cold days^5,6^. In line with rising temperatures, Iran experiences a significant rise in aridity and evaporation^7,8^, but also in extreme hydroclimatic events such as floods and droughts^9,10^. Chaparinia et al.^11^ reported significant reductions in heavy precipitation indices and total wet-day precipitation across many regions of Iran—particularly in the drier areas—accompanied by a marked increase in consecutive dry days. These spatially differentiated trends point to an overall shift toward greater water scarcity, especially in already arid parts of the country. Most climate projection studies conclude that large parts of Iran are experiencing a trajectory toward warmer and drier climate conditions, with the most pronounced impacts expected in already fragile semi-arid regions^6,12^. Drought duration, intensity, and frequency are expected to increase significantly^13,14^, especially in the eastern and northeastern parts of the country^15,16^. However, long-term instrumental climate records in Iran are limited in both duration and spatial coverage, particularly in high-elevation and remote regions, which constrains the assessment of long-term hydroclimatic variability and highlights the need for proxy-based reconstructions.

Although numerous investigations have assessed drought trends in Iran using different indicators—including Standardized Precipitation Index (SPI), Standardized Precipitation Evapotranspiration Index (SPEI), and Palmer Drought Severity Index (PDSI)—most focus on general water deficits without distinguishing the underlying drivers or their different impacts on ecosystems. To date, no study has examined long-term changes in vapor pressure deficit (VPD), a key atmospheric metric of moisture demand, despite its critical role in shaping drought intensity under warming conditions. Drought itself can manifest in different forms depending on the variable of interest and spatial or temporal scale, such as meteorological, hydrological, agricultural, and socioeconomic droughts^17,18^. From an eco-physiological perspective, drought can arise from soil moisture–related drought conditions (as approximated by SPEI, which integrates precipitation and atmospheric demand) or from atmospheric moisture deficits (as reflected by VPD), each imposing stress on vegetation^19^.

With ongoing climate change, mountain forests and woodlands in Iran are increasingly exposed to severe droughts and elevated atmospheric moisture deficits, reducing resilience and increasing vulnerability of these ecosystems. According to projections of the future drought vulnerability index, current areas classified as having very low vulnerability are projected to shift toward very high vulnerability, particularly in eastern and northeastern Iran^20^, underscoring the importance of studying the impacts of drought on regional ecosystems. Trees may be affected by soil drought, limiting water uptake, and by atmospheric drought, which drives evaporative demand. Due to expected increases in drought intensity, frequency, and duration, understanding which type of drought most strongly constrains tree growth and vitality is essential for anticipating regional forest responses and managing ecosystem risks. However, our current understanding of how soil versus atmospheric drought shapes tree physiology and growth is still limited. Long-term records that separately capture these different aspects of drought are therefore urgently needed.

Dendroclimatology offers a valuable approach to investigating historical tree responses to drought by using annually resolved records preserved in tree rings^21–23^. In Iran, tree-ring studies have provided insights into past hydroclimatic variability^24–27^. While some studies have assessed general drought variability using tree rings^28,29^, no work has yet distinguished between soil and atmospheric drought parameters using tree-ring parameters, nor evaluated their respective impacts on tree growth in Iran. Long-lived *Juniperus polycarpos* are particularly suitable for disentangling drought impacts, given their multi-centennial lifespans and pronounced sensitivity to moisture fluctuations^25,30^. This distinction is particularly important given that tree-ring width (TRW) predominantly reflects growth processes tied to carbon assimilation and water availability in soils^31,32^, while stable oxygen isotopes (δ^1^⁸O) in cellulose primarily record the δ^1^⁸O signal of source water adjusted during evaporative enrichment at the leaf level, which is linked to atmospheric demand and stomatal regulation^33,34^. Under arid climate conditions, δ^1^⁸O in tree rings has proven to be a promising proxy for recording variations of precipitation, relative humidity, or VPD^35,36^, rather than primarily reflecting the isotopic signal of the source water^37–39^. This divergence in the δ^1^⁸O signal preserved in tree rings is determined mainly by the extent of isotopic exchange between source water and phloem sugars during cellulose synthesis—a physiological process that is modulated by site conditions^40–42^. Accordingly, tree-ring δ^1^⁸O has been widely used as a tool for reconstructing hydroclimate variability^21,43,44^. Foroozan et al.^26^ found that δ^1^⁸O in junipers in northern Iran is a robust proxy for atmospheric moisture deficit and reconstructed precipitation changes and drought episodes for the past 500 years^25^.

In this study, we utilize multi-centennial TRW and δ^1^⁸O chronologies from long-lived *J. polycarpos* in northeastern Iran to reconstruct both soil drought (SPEI) and atmospheric drought (VPD) over the past two centuries using multi-proxy regression models. This dual-reconstruction approach enables us to assess the relative contributions of soil versus atmospheric moisture deficits to tree growth reductions—a distinction not previously explored in Iranian dendroclimatology. Our results provide novel insights into how different types of drought stress constrain semi-arid forest ecosystems under a changing climate.

## Materials and methods

### Study area and regional climate

The Hezar Masjed Mountains (HM) are located in northeastern Iran and stretch for approximate.

ly 500 km in a northwest–southeast (NW–SE) direction forming part of the northern highlands of Khorasan Razavi Province. The HM range acts as a natural barrier for moist air masses, separating the arid Qara Qum Desert to the north from the Mashhad plains to the south.

**Fig. 1:**
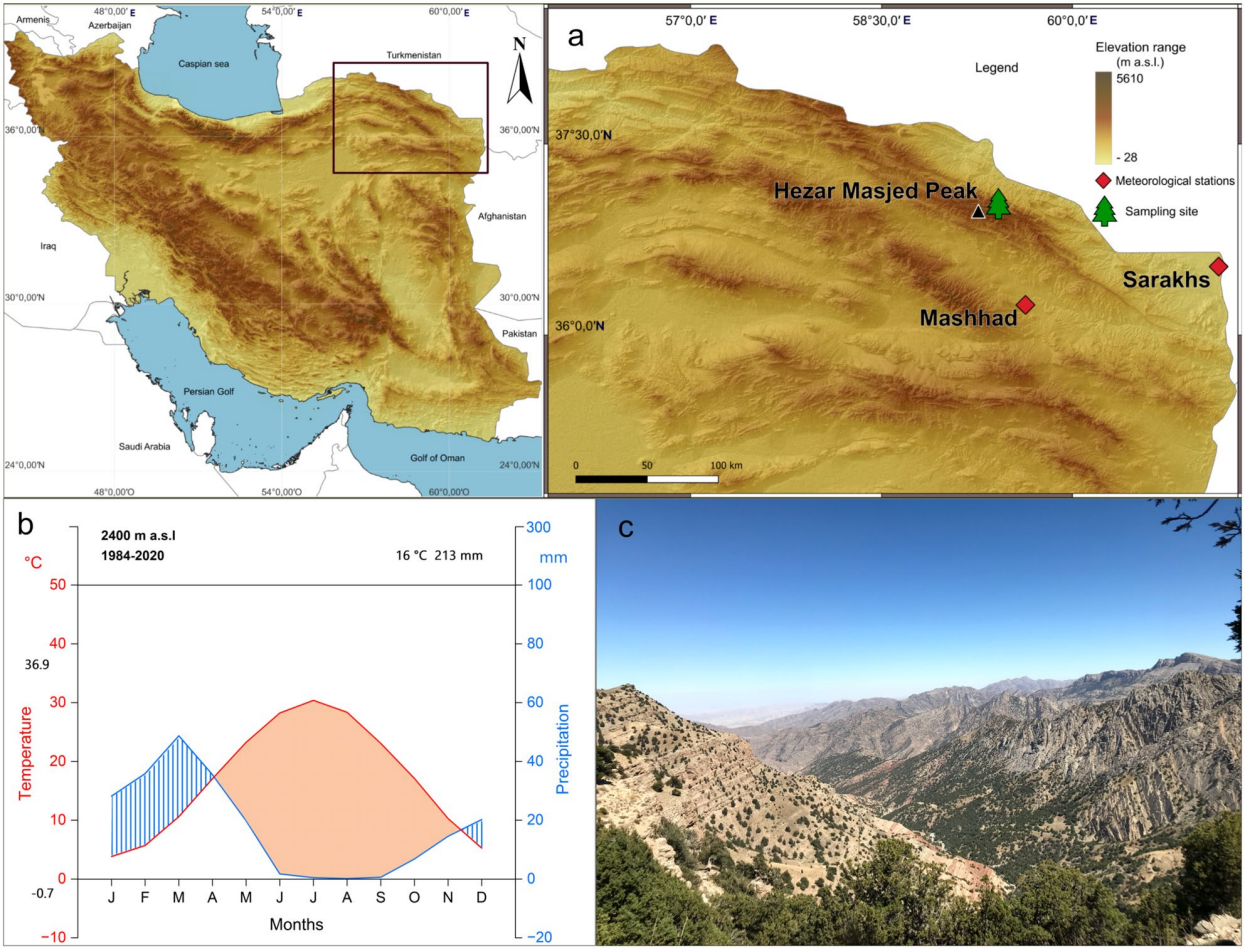
(**a**) Location of the study site in the Hezar Masjed Mountains, northeastern Iran. The map was created using QGIS (version 3.44.9; https://www.qgis.org). Elevation data were derived from a digital elevation model (DEM) obtained from the USGS EarthExplorer platform (https://earthexplorer.usgs.gov). (**b**) Ombrothermic diagram illustrating the monthly mean temperature (red line) and precipitation (blue line) recorded at the Hezar Masjed station (2400 m a.s.l.) for the period 1984–2020. (**c**) Landscape view of the Hezar Masjed Mountains showing a typical high-elevation juniper forest (photograph taken by the authors).

The highest peak, Mount Hezar Masjed (36°58′12″N, 59°21′36″E; 36.97311°N, 59.35612°E) (Fig. 1), rises to approximately 3,130 m above sea level (m a.s.l.) and is located about 70 km northwest of Mashhad. Phytogeographically, the region belongs to the Irano-Turanian zone and is characterized by open juniper woodlands typical for mountain forest-steppe ecosystems on north-facing as well as on south-facing slopes^45^. Juniper trees grow from elevations as low as 1,340 m but form denser stands between 2,000 and 2,700 m a.s.l^46^. The soils in these habitats are shallow, calcareous, and alkaline, with pH values ranging from 7.7 to 8.7, with limited horizon development and low organic matter^46^. According to the FAO national soil map of Iran^47,48^, the dominant soil types in the region are calcareous lithosols, brown soils, and chestnut soils, with surrounding areas classified as brown soils–lithosols. These classifications indicate predominantly shallow, stony soils over limestone bedrock, with limited profile development and low organic matter. Based on the World Reference Base (WRB), these soils correspond primarily to Leptosols and Regosols, with possible Calcisols in calcareous zones. These soil types are typical of arid and semi-arid mountain ecosystems and are characterized by low water retention capacity.

The regional bioclimate is Mediterranean xeric-continental,^49^, characterized by pronounced seasonality, high continentality, and dry, warm summers. The wet season lasts 5 to 6 months, spanning mid-autumn (mid-October) through spring (May), with its peak precipitation occurring in winter (Fig. 1b). In contrast, the summer months (July to September) are dry, with elevated VPD and high evapotranspiration, creating stressful conditions under which only cold- and drought-tolerant species such as juniper can persist. The thermal growing season likely spans from March to September, based on seasonal temperature trends, although no studies have yet documented the precise timing of cambial activity in *Juniperus polycarpos* for the region. Our sampling site is situated in the southern part of the Hezar Masjed Mountains in Razavi Khorasan Province, NE Iran, at elevations between 2000 and 2700, corresponding to the core zone of juniper forest distribution (Fig. 1).

### Tree species and sampling

The tree-ring samples were collected from an open *J. polycarpos* forest stand located at 37°02′–03′ N and 59°18′–24′ E. Our sampling strategy focused on selecting mature trees of dominant size and healthy appearance to maximize the length of the tree-ring chronology and minimize the occurrence of false or missing rings. Species identification of *J. polycarpos* was verified by experts from the Faculty of Natural Resources, University of Tehran, during field sampling. Reference voucher material from the study site is deposited in the plant and wood collection of the Faculty of Natural Resources, University of Tehran. Sampling permissions and licenses for collecting tree cores were obtained prior to the field campaigns in accordance with national regulations governing scientific field studies.

Due to the site’s popularity among local hikers and campers, it was challenging to find old-growth trees undisturbed by human activity. Therefore, we collected samples from trees growing across a range of elevations, between 2,000 and 2,656 m a.s.l from locations where human impact was minimal. This elevation variability among sampled trees was later considered in the isotope analysis to account for potential altitudinal effects on δ^1^⁸O values. We also ensured that the sampled trees were not accessing groundwater, to avoid interference with the climate signal recorded in δ^1^⁸O values. We extracted at least two increment cores at breast height (ca. 1.3 m), approximately 180° apart around the stem, from each of 47 living *J. polycarpos* trees, using a 5 mm increment borer.

### Tree-ring width measurement and chronology development

All increment cores (n = 98) were air-dried and prepared by sanding the surface with progressively finer sandpaper to clearly reveal annual ring boundaries, following standard procedures^50^. Tree-ring widths were measured under a stereomicroscope using the LINTAB 6 measuring system^51^, a precision of 0.001 mm. Visual and statistical cross-dating was conducted within and between trees to assign each annual ring to the exact calendar year. Cross-dating accuracy was verified using TSAP-Win™ software^52^, based on statistical parameters such as Gleichläufigkeit (GLK) and t-values^53,54^. This process also helped identify and correct false or missing rings, as well as minimize measurement errors. To develop the site chronology, individual tree-ring series were detrended to remove biological age-related growth trends using a signal-free age-dependent spline detrending approach^55^, implemented in the RCSigFree_45v2b software (www.ldeo.columbia.edu). This approach removes biological growth trends while minimizing distortion of any long‐term climatic variance in the final chronology. The chronology was developed by calculating a bi-weight robust mean of all detrended and cross-dated individual series.

The chronology quality and strength of the common signal between the individual tree-ring series were assessed by computing expressed population signal (EPS), sub-sample signal strength (SSS), mean inter-series correlation (Rbar), signal-to-noise ratio (SNR), and first-order autocorrelation. These were calculated using the dplR package^56^ in R^57^. Although the full tree-ring width chronology spans 499 years (1523–2021), the present analysis focuses on the common period from 1821 to 2020, which overlaps with the stable isotope record and allows for integrated multi-proxy analysis.

### Stable oxygen isotope analysis

For stable isotope analysis, we selected trees that exhibited strong growth coherence with the mean local tree-ring width chronology and showed no evidence of missing or false rings. In line with standard recommendations^33,58^, we ensured a minimum replication of five individual trees throughout the entire period of investigation (1821–2020), allowing the construction of a robust mean isotope chronology representative of regional climate signals. Individual annual rings for the selected trees were carefully separated using a scalpel, then split into smaller fragments and stored in Eppendorf tubes for α-cellulose purification. The α-cellulose component was isolated using a multi-stage chemical procedure described by^59^, which included pretreatment, bleaching, and purification steps. The purified α-cellulose was homogenized using an ultrasonic device to ensure thorough mixing of earlywood and latewood components from each annual ring. This homogenization step is critical because it integrates the full climate signal captured within a given year. The homogenized samples were then freeze-dried prior to isotope analysis.

Dried α-cellulose samples were weighed into silver capsules and analyzed for stable oxygen isotope ratios (δ^1^⁸O) using a high-temperature pyrolysis system (HT Oxygen Analyzer) coupled to a continuous-flow isotope ratio mass spectrometer (IRMS; Delta V Advantage, Thermo Fisher Scientific). All analyses were performed at the Stable Isotope Laboratory of the Institute of Geography, Friedrich-Alexander-Universität Erlangen-Nürnberg. Analytical precision was typically better than ± 0.25‰ for δ^1^⁸O measurements. The oxygen isotope values are expressed as δ^1^⁸O in per mil (‰) relative to the international VSMOW standard (Vienna Standard Mean Ocean Water).

### Climate data and drought index acquisition

Due to the remote and mountainous location of the study area, no meteorological station is located directly within the sampling site. This limitation is common in remote mountainous regions of northeastern Iran, where long-term high-elevation climate records are largely unavailable. While absolute climatic conditions may differ with elevation, nearby stations are assumed to capture regional variability in temperature and moisture conditions. Therefore, we collected and reviewed all available instrumental data from surrounding areas. Among these, two synoptic stations, Mashhad and Sarakhs, provided the most consistent records and strongest correlations with both tree-ring parameter variables. Therefore, the arithmetic mean of their data was used to approximate regional-scale climate variability despite elevation-related differences in absolute values and to serve as input for the calculation of VPD.

For the calibration period (1984–2020), the mean annual precipitation was 213 mm, and the mean annual temperature was 16.93 °C (Fig. 1). The driest period during the year extends from June to September, with August receiving the lowest monthly precipitation (0.19 mm). The wettest months occur during winter and early spring, with a maximum in March (48.7 mm). Mean monthly temperatures range from 3.8 °C in January to 28.4 °C in August (Fig. 1). To quantify atmospheric drought, we calculated vapor pressure deficit (VPD) using monthly maximum temperature and relative humidity data averaged from the Mashhad and Sarakhs synoptic stations. VPD was computed using standard equations that calculate the difference between saturation vapor pressure and actual vapor pressure^60^. Monthly VPD values were averaged to characterize seasonal atmospheric dryness and were used as an indicator of the evaporative demand imposed on trees.

Besides instrumental climate data, we used the Standardized Precipitation Evapotranspiration Index (SPEI) at multiple timescales (SPEI01–SPEI07), which integrates both precipitation and potential evapotranspiration (PET) to assess hydroclimatic drought conditions related to soil moisture availability. We focused on short-term accumulation periods (SPEI01–SPEI07), as these are most relevant to growing-season physiological processes and showed the strongest and most consistent relationships with the tree-ring proxies.

Monthly SPEI data were obtained from the Global SPEI Database (https://spei.csic.es/spei_database), which provides global-scale drought information at 0.5° spatial and monthly temporal resolution, based on precipitation and PET data from the Climatic Research Unit (CRU) of the University of East Anglia. Data were extracted for the grid cell that best covers the coordinates of our study area. Given the coarse spatial resolution of the CRU-based SPEI product and the mountainous terrain of the study area, these data are interpreted as representing regional hydroclimatic variability rather than exact local site conditions.

### Statistical analyses

All statistical analyses were conducted in R (version 4.5.3)^57^. Specific packages used for individual analyses are described below, where relevant.

### Altitude correction of δ^1^⁸O values

The sampled *J. polycarpos* trees were distributed across a wide elevational gradient (approximately 2000 to 2656 m a.s.l.). To assess whether tree-ring δ^1^⁸O values were influenced by elevation, we tested for a significant altitude effect^61,62^. Such an effect has been reported in previous studies e.g.,^63–65^, typically reflecting changes in temperature, precipitation isotopic composition, and VPD along elevation gradients. In particular, the δ^1^⁸O of source water generally decreases with increasing elevation due to isotopic fractionation during precipitation, while changes in temperature and evaporative demand can further influence δ^1^⁸O enrichment in leaf water during cellulose synthesis^66–68^. A linear regression model was applied to the mean δ^1^⁸O value of each tree over the study period against its corresponding sampling elevation:

1 $$\delta^{18} {\mathrm{O}} = {\upbeta}_{0} + {\upbeta}_{1} \cdot {\mathrm{Elevation}}$$

The regression revealed a statistically significant negative relationship between elevation and δ^1^⁸O (r = 0.75, p < 0.01), corresponding to a decrease of 0.339‰ per 100 m of elevation gain. The model explained 56.4% of the variance in δ^1^⁸O values (R^2^ = 0.564), supporting a moderate but significant altitudinal effect, which is illustrated in Supplementary Fig. S1. Based on this relationship, δ^1^⁸O values were normalized to a reference elevation of 2000 m a.s.l. for all trees using the equation:

2 $$\delta^{18} {\mathrm{O}}_{corrected} = \delta^{18} {\mathrm{observed}} + {\upbeta}_{1} \cdot \left( {2000 - {\mathrm{Elevation}}} \right)$$

This correction was applied to improve comparability among trees sampled across different elevations and to reduce potential altitudinal bias in the isotope–climate signal, while preserving the temporal variability of the individual series (Fig. S2). Because this approach assumes that the elevation effect remains temporally stable, it is acknowledged as a potential limitation.

The corrected δ^1^⁸O values were used in all subsequent statistical analyses, including climate response analysis and reconstruction.

### Correlation analyses and climate reconstruction using tree-ring parameters

To reconstruct seasonal drought indices, we evaluated the relationships between TRW, corrected δ^1^⁸O values, and the soil and atmospheric drought indicators. Specifically, we assessed correlations between tree-ring parameters and SPEI indices across accumulation periods ranging from one to seven months (SPEI01–SPEI07), as well as across individual calendar months. All correlation analyses using Spearman rank correlations with bootstrapped significance testing were performed using the dendroTools package^69^. Monthly and seasonal VPD values were examined similarly using both tree ring proxies. To determine the best predictor(s) for reconstructing seasonal drought variability, we used linear regression models. Separate models were built for (1) VPD using δ^1^⁸O as the sole predictor, and (2) SPEI using both TRW and δ^1^⁸O as predictors. In the latter case, we employed multiple linear regression to test whether combining both proxies, which capture partly distinct drought-related processes, improved reconstruction skill relative to single-proxy models. The target variable for the soil drought reconstruction was SPEI07 for September, selected based on its strong and significant correlations with both proxies and its ability to represent cumulative growing-season moisture conditions. Longer accumulation periods may better reflect long-term hydrological drought persistence, but they are less directly linked to growing-season physiological processes governing annual ring formation and were therefore not the focus of this study. The VPD reconstruction target was the mean March–September VPD, which correlated most strongly with δ^1^⁸O.

To identify the most effective predictor or combination of predictors for reconstructing seasonal drought variability, we performed both simple and multiple linear regression analyses. Prior to model fitting, we assessed the underlying assumptions of linear regression, including linearity, homoscedasticity, and normality of residuals. Predictor collinearity for the multiple regression model was tested using the variance inflation factor (VIF), and found to be below the commonly accepted threshold (VIF < 5), indicating acceptable levels of multicollinearity.

Given the relatively short instrumental period (1984–2020), leave-one-out cross-validation (LOOCV) was employed to evaluate the temporal robustness and predictive accuracy of each model. For each iteration, one year was withheld from the training dataset, the model was re-fit using the remaining years, and the withheld year was predicted. This process was repeated across all years to calculate cross-validated performance metrics, including the coefficient of determination (R^2^), root mean square error (RMSE), mean absolute error (MAE), and the reduction of error (RE).

The final models were applied to the full tree-ring dataset (1821–2020) to generate annually resolved reconstructions of SPEI and VPD.

### Classification of drought years and growth response

To classify drought years in a way that reflects the specific climatic conditions and tree growth responses in our study region, we applied K-means clustering to the reconstructed time series of vapor pressure deficit (VPD), Standardized Precipitation-Evapotranspiration Index (SPEI), and a combined matrix of both indices, spanning the period 1821–2020. All classifications are based on reconstructed growing-season drought conditions (March–September), consistent with the temporal scale of the climate reconstructions. Thus, references to “drought years” throughout the manuscript refer to years characterized by drought conditions during the growing season rather than annual means.

These classifications were used to distinguish between different types of droughts: VPD-only drought, SPEI-only drought, and combined drought years (COMB) to reflect years with either co-occurring or non-coinciding atmospheric and soil moisture deficits. For each input (VPD, SPEI, and combined), the number of clusters was set to three (k = 3), corresponding to years of severe drought, moderate drought, and non-drought conditions. This unsupervised, data-driven approach identifies natural groupings in the dataset by minimizing within-cluster variance and offers an ecologically meaningful classification that avoids the limitations of fixed percentile thresholds (e.g., using SPEI thresholds such as SPEI <  − 2 for extreme drought). As a result, the drought categories better capture the site-specific hydroclimatic regimes that trees have experienced historically.

Years assigned to the lowest, intermediate, and highest value clusters were labeled as severe drought (D), moderate drought (M), and non-drought (ND), respectively, for each index. This resulted in three classes per index (e.g. VPD-D, VPD-M, VPD-ND). The table below summarizes the classification scheme (Table 1):

**Table 1:** Classification scheme used for drought year (based on growing-season conditions) identification based on cluster analysis of reconstructed drought indices.

Cluster level	Label	Abbreviations
Individual drought indices (VPD or SPEI)		
Severe drought	-D	VPD-D/SPEI-D
Moderate drought	-M	VPD-M/SPEI-M
No drought	-ND	VPD-ND/SPEI-ND
Combined VPD and SPEI clusters		
Combined severe drought	-D	COMB-D
Combined moderate drought	-M	COMB-M
Combined non-drought	-ND	COMB-ND

This clustering-based classification scheme was then used to test growth sensitivity to different drought types.

We applied Superposed Epoch Analysis (SEA) using the dplR package^56^ to assess how tree growth responded to these distinct drought types. SEA was conducted using the tree-ring width index chronology (RWI), with severe drought years identified from each cluster (VPD-D, SPEI-D, COMB-D). For each drought type, SEA was performed using a ± 3-year lag window around the drought event (lag 0), and 1000 Monte Carlo resampling iterations were used to generate confidence intervals and assess statistical significance. This approach allowed us to test whether tree growth significantly deviated from the long-term mean in the drought year and surrounding years, offering insights into both the immediate and legacy effects of drought on growth.

## Results

### Climate sensitivity of tree-ring parameters

**Fig. 2:**
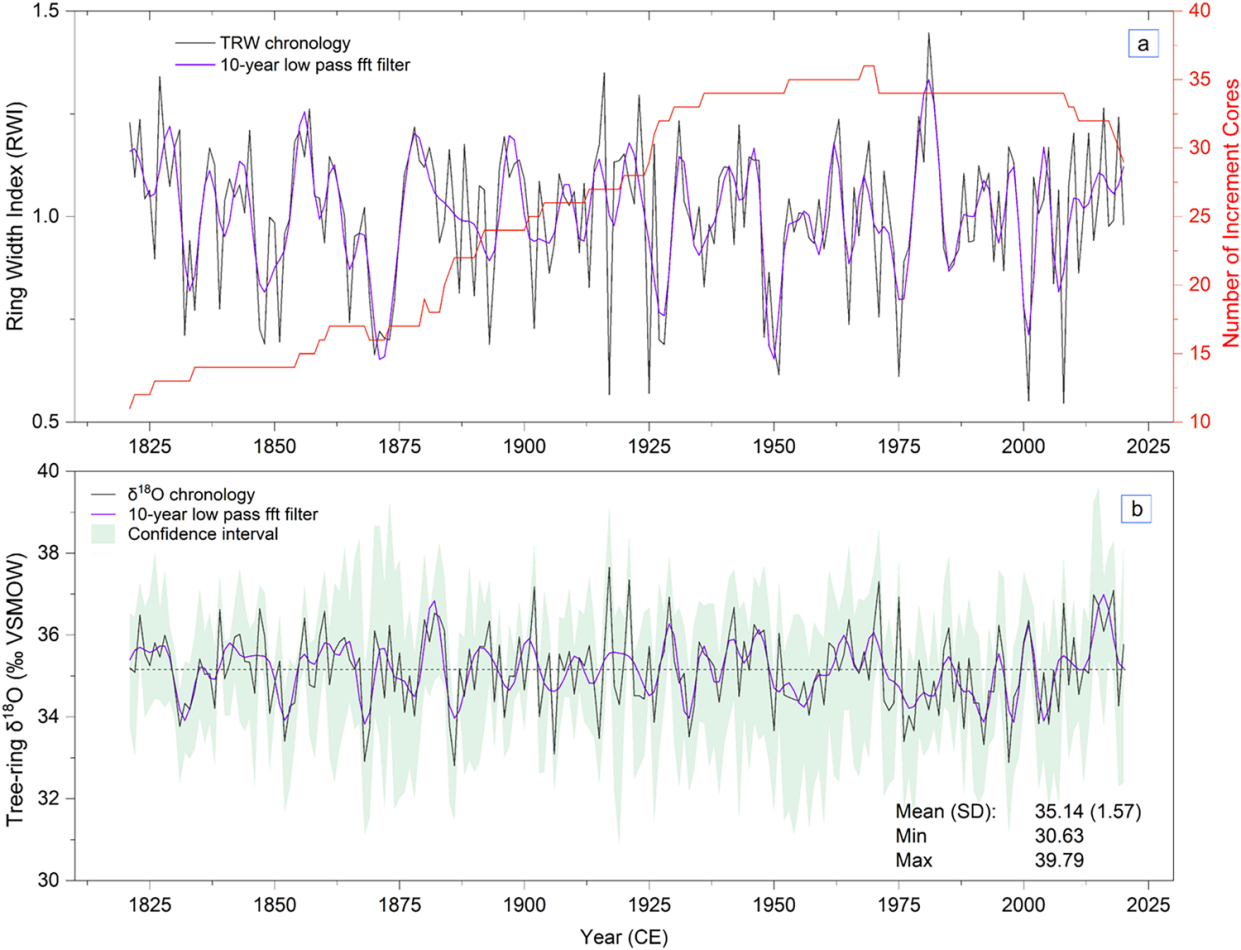
Tree-ring width index (RWI) and stable oxygen isotope (δ^1^⁸O) chronologies of J. polycarpos from the Hezar Masjed Mountains, NE Iran, from 1821 to 2020. (**a**) Standardized ring width index (RWI) with 10-year low-pass FFT filter (purple) and sample depth (number of increment cores, red). (**b**) δ^1^⁸O chronology with 10-year FFT filter and 95% confidence interval (green shading).

Figure 2 indicates the TRW and δ^1^⁸O chronologies developed from *J. polycarpos* cover the period 1821–2020. The TRW chronology exhibited higher temporal autocorrelation (0.354) than δ^1^⁸O (0.144) (Table 2), reflecting a strong biological memory. Inter-series correlations were also high for both proxies (r̄ = 0.322 for RWI; 0.490 for δ^1^⁸O), indicating a strong common signal among trees. Both chronologies were well replicated and exceeded the widely accepted thresholds of climate sensitivity, with EPS values > 0.88, SSS > 0.89, and high signal-to-noise ratios (7.70 for δ^1^⁸O, 8.60 for RWI). Both chronologies display clear inter-annual variability as well as multi-decadal fluctuations over the common period (1821–2020), reflecting coherent yet contrasting responses of growth and isotopic composition to long-term environmental and climatic variability in the region (r =  − 0.28, p < 0.001).

**Table 2:** Statistical characteristics of the tree-ring width index (RWI) and stable oxygen isotope (δ^1^⁸O) chronologies of J. polycarpos.

Descriptive statistics of tree ring chronology	1821–2020	
	δ^1^⁸O	RWI
Inter-series correlation (r bar)	0.490	0.322
First-order autocorrelation (AC1)	0.144	0.354
Expressed population signal (EPS)	0.885	0.895
Subsample signal strength (SSS)	0.929	0.891
Signal-to-noise ratio (SNR)	7.698	8.562

We assessed the correlations between δ^1^⁸O, RWI, and both VPD and SPEI indices across monthly and seasonal scales, including different SPEI accumulation periods (SPEI01–SPEI07), during the calibration period (1984–2020).

**Fig. 3:**
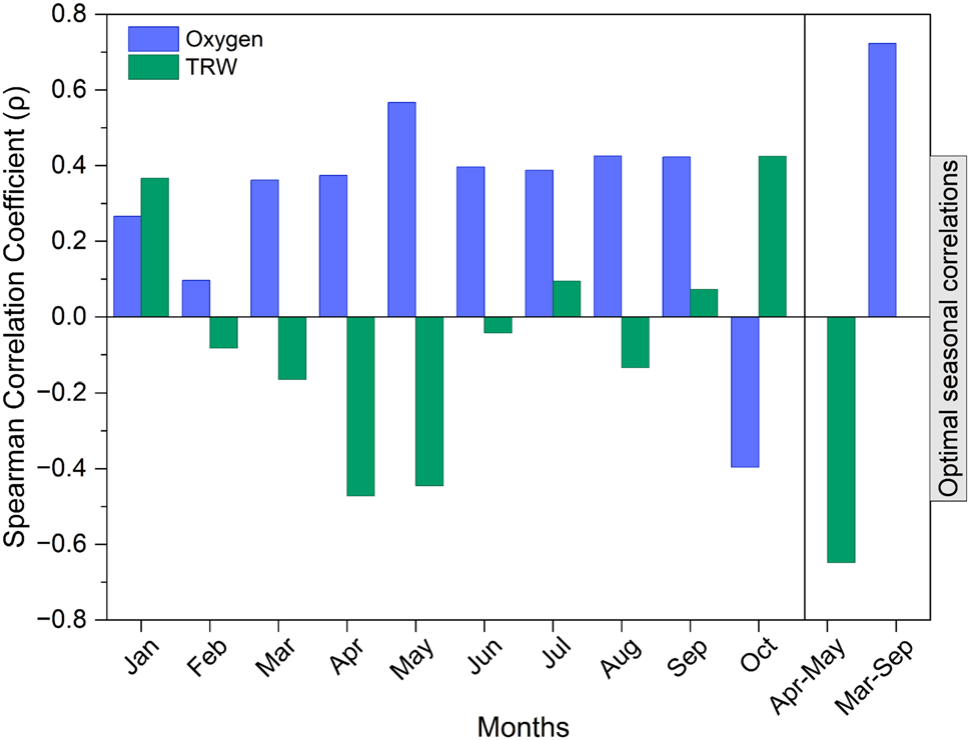
Bootstrapped Spearman rank correlations (ρ) between various monthly and seasonal vapor pressure deficit (VPD) values and the two tree-ring parameter series (RWI, δ^1^⁸O) during the calibration period (1984–2020).

The analyses revealed a clear difference in climate sensitivity between the two parameters. As shown in Fig. 3, δ^1^⁸O exhibited positive and significant correlations with monthly VPD from March through September, peaking in May (ρ = 0.575). Seasonal correlations further confirmed its sensitivity to atmospheric moisture demand, with the highest correlation for the March–September mean VPD (ρ = 0.72). In contrast, TRW showed weaker and generally negative correlations with VPD from April to June, with the strongest negative correlation in April–May (ρ = − 0.64). Based on these results and the seasonal dynamics of tree physiology in the study region, we selected VPD (March–September) to represent atmospheric moisture conditions during the full growing season for reconstructing atmospheric drought.

**Fig. 4:**
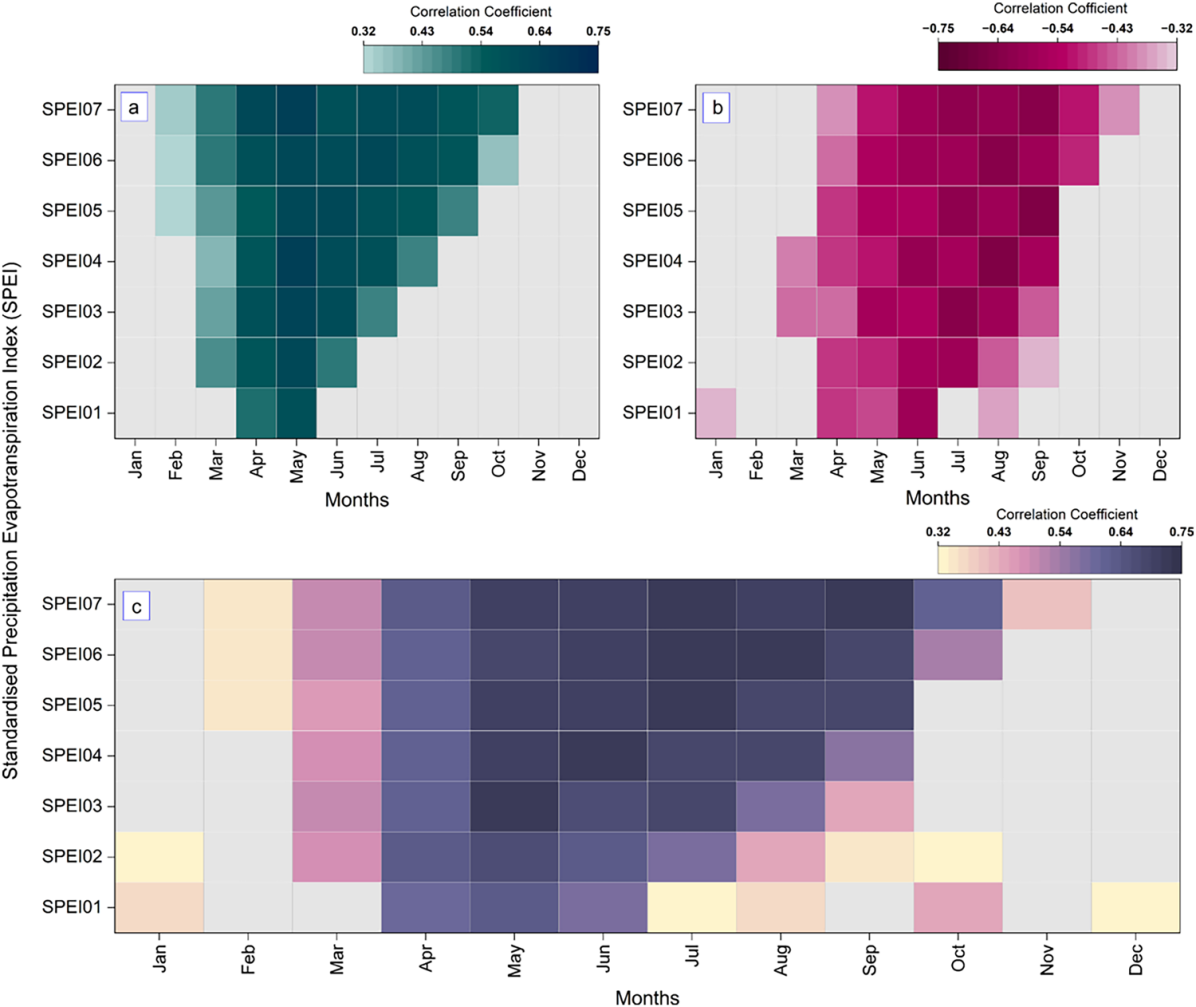
Correlation heatmaps of monthly Standardized Precipitation Evapotranspiration Index (SPEI) with tree-ring proxies and combined models. (**a**) TRW-SPEI correlations, (**b**) δ^1^⁸O-SPEI correlations, and (**c**) combined model (TRW + δ^1^⁸O) correlations with SPEI. Each panel shows correlations for SPEI accumulation periods (SPEI01–SPEI07) across months.

Results of the correlation analyses with SPEI are shown in Fig. 4. Correlation strengths varied between proxies, accumulation periods, and months. δ^1^⁸O showed moderate to strong negative correlations with SPEI, especially for accumulation periods of 5 to 7 months during late spring and summer (Fig. 4b). The strongest monthly relationship was observed with SPEI05 in September (ρ = − 0.653), whereas the whole growing season (March-September) target variable, SPEI07 in September, also showed a significant correlation with δ^1^⁸O(ρ = − 0.617).

In contrast, RWI response to shorter accumulation windows earlier in the growing season, with the highest correlation for SPEI04 in May (ρ = 0.652; Fig. 4a), while the correlation with SPEI07 in September remained significant but lower (ρ = 0.565).

Combining both proxies in a multiple linear regression model further improved the correlation with seasonal SPEI, confirming their complementary value. the strongest multiple correlation was found for SPEI05 in July (r = 0.73), while SPEI07 in September also showed a strong relationship (r = 0.715; Fig. 4c).

Although some shorter accumulation periods and narrower monthly windows showed slightly stronger correlations with individual proxies (Table S1), SPEI07 in September was selected as the reconstruction target because it best represents the cumulative March–September moisture balance over the full growing season and therefore aligns most closely with the physiological period relevant to annual ring formation at the study site.

Accordingly, SPEI07 September was chosen as the soil drought target for reconstruction using the combined TRW and δ^1^⁸O predictors, while growing-season VPD (March–September) was used to represent atmospheric drought, reconstructed using δ^1^⁸O alone.

### Tree-ring based drought reconstructions

#### VPD reconstruction

To reconstruct atmospheric drought, we developed a simple linear regression model using elevation-corrected δ^1^⁸O values as the predictor of growing-season VPD (March–September mean). The final model was defined as:

3 $${\text{VPD }} = \, {-}{ 4}.{643 } + \, 0.{218}\cdot\delta^{18} {\mathrm{O}}$$

**Fig. 5:**
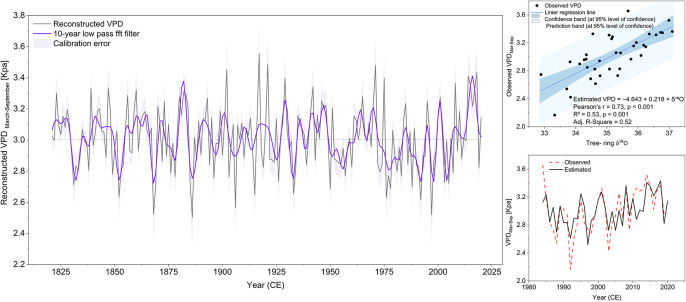
Reconstruction of growing-season atmospheric drought (VPD March–September) based on δ^1^⁸O from J. polycarpos. Left: Reconstructed VPD (black) with 10-year low-pass FFT filter (purple) and calibration error as grey shading. Top right: Calibration scatter plot of observed vs predicted VPD with regression line, 95% confidence band, and prediction interval. Bottom right: Time series comparison of observed and predicted VPD during the calibration period (1984–2020).

The model explained 53.4% of the variance in observed VPD (R^2^ = 0.534, Adj. R^2^ = 0.520, *p* < 0.001), with a residual standard error of 0.22 kPa (Fig. 5, right panel). The Durbin–Watson test indicated some degree of autocorrelation in residuals (DW = 1.38, *p* = 0.022), a typical feature in dendroclimatological time series^70,71^. Leave-One-Out Cross-Validation (LOOCV) confirmed the robustness of the model, with RMSE = 0.22, MAE = 0.17, and cross-validated R^2^ = 0.48. In addition, the Reduction of Error (RE) was calculated as 0.48, indicating that the model performs substantially better than the climatological mean and provides skillful year-to-year predictions across the calibration period (1984–2020). The final model was used to estimate VPD from 1821 to 2020, resulting in a high-resolution reconstruction of growing-season atmospheric moisture demand over two centuries (Fig. 5, left panel). The reconstruction reveals both short-term peaks and multi-decadal fluctuations in VPD, with notable dry periods occurring in the mid-1800s, the early 1900s, and the post-1980s period. A 10-year FFT low-pass filter highlights long-term atmospheric drying trends in the twentieth century.

Before proceeding to the soil drought reconstruction, we evaluated the effect of the applied altitude correction on δ^1^⁸O. We compared model performance and correlations using the uncorrected and corrected δ^1^⁸O chronologies. The corrected δ^1^⁸O series showed stronger correlations with both VPD (r = 0.73 vs. 0.70) and SPEI (r = –0.63 vs. –0.60), and slightly improved regression model performance (Adj. R^2^ = 0.52 for VPD; 0.48 for SPEI) compared to the uncorrected series (Adj. R^2^ = 0.47 for both). These results confirm that elevation correction enhances the climatic signal preserved in the δ^1^⁸O chronology and improves the predictive skill of the drought reconstructions, while preserving the temporal variability and coherence among individual series (Figure S2), indicating that the correction does not distort the underlying climatic signal.

#### SPEI reconstruction

Further, we developed a multiple linear regression model using elevation-corrected δ^1^⁸O values and tree-ring width index (RWI) as predictors of SPEI07 for September to reconstruct growing-season soil moisture drought.

The final model was defined as:

4 $${\text{SPEI }} = { 14}.{371 }{-} \, 0.{491}\cdot\delta^{18} {\text{O }} + { 2}.{537}\cdot{\mathrm{RWI}}$$

Both predictors were statistically significant (p < 0.01), with δ^1^⁸O showing a negative relationship and TRW a positive one, consistent with their known physiological responses to water availability. The model explained 51.1% of the variance in observed SPEI values (R^2^ = 0.511, Adj. R^2^ = 0.483) and had a residual standard error of 0.78 (Fig. 7, right panel). Model assumptions were met, although the Durbin-Watson test indicated potential autocorrelation in residuals (DW = 1.37, *p* = 0.021), a common feature in climate time series. Model validation using Leave-One-Out Cross-Validation (LOOCV) demonstrated robust predictive skill, yielding an average RE (Reduction of Error) of 0.98, with an RMSE = 0.81 and MAE = 0.67. The LOOCV R^2^ was 0.43, indicating good model stability across the calibration period (1984–2020).

**Fig. 6:**
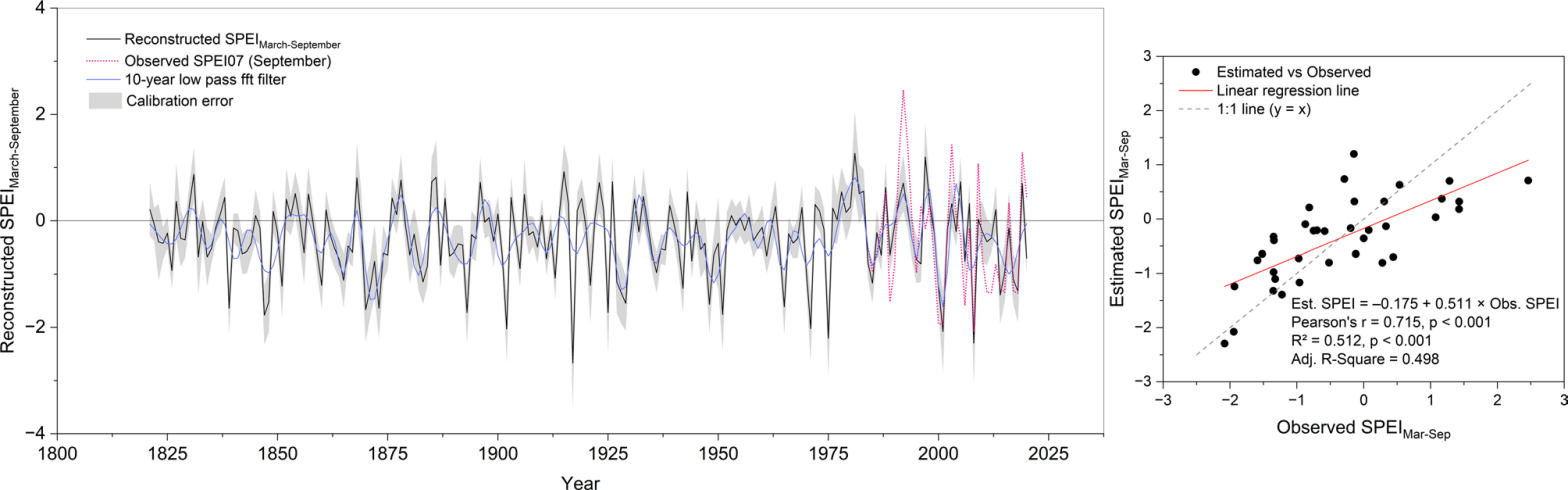
Reconstruction of the growing-season SPEI based on δ^1^⁸O and TRW parameters from J. polycarpos. Left Panel: Reconstructed (black) and observed (pink dashed) SPEI07 September values with a 10-year low-pass FFT filter (blue). The shaded area shows ± 1 standard error of model residuals during calibration. Right: Calibration plot comparing observed versus estimated SPEI values for the period 1984–2020. The solid red line indicates the best-fit linear regression between observed and predicted SPEI values (R^2^ = 0.51, p < 0.001), while the dashed gray line represents the 1:1 line (y = x), indicating perfect agreement.

The final model was applied to the full proxy record to produce an annually resolved reconstruction of SPEI07 for September spanning the period 1821–2020 (Fig. 6, left panel). The reconstruction captures high interannual and decadal variability in soil moisture availability, with extended droughts evident in the late nineteenth century and 20th-century multi-year dry phases. A 10-year low-pass FFT filter highlights longer-term hydroclimatic trends. The 10-year low-pass FFT–filtered reconstructions of VPD and SPEI are shown together in Supplementary Figure S3, highlighting their coherent decadal-scale variability over the past two centuries. During the calibration period (1984–2020), observed VPD and SPEI exhibited a significant negative correlation (r = − 0.58, p < 0.001), consistent with the expected inverse relationship between atmospheric demand and soil moisture availability. At decadal timescales, the smoothed reconstructed series also showed a significant negative correlation (r = − 0.69, p < 0.001), although some deviations between the series were evident.

### Classification of drought events

**Fig. 7:**
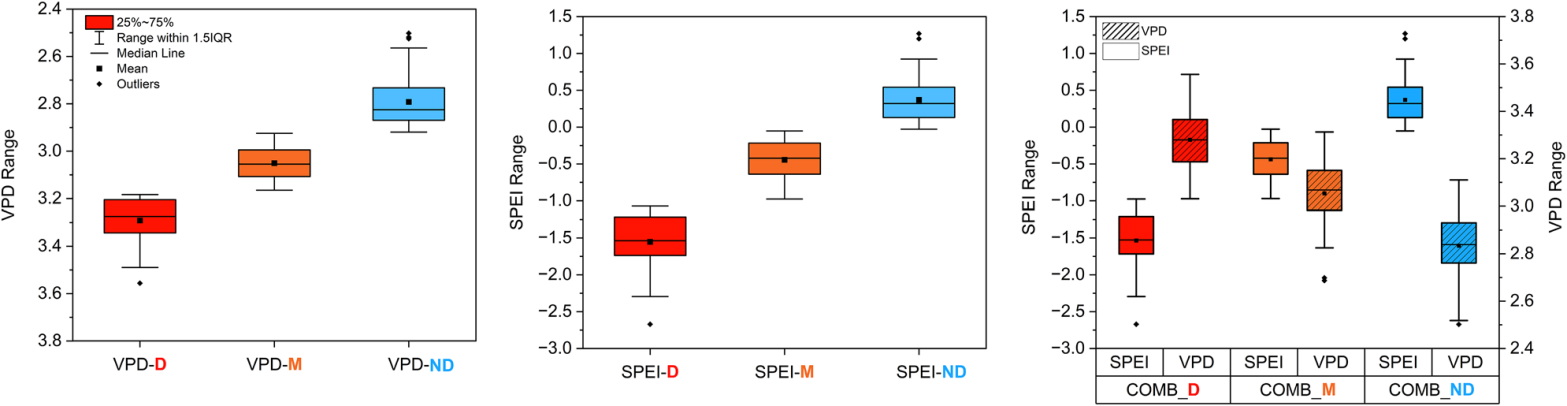
Boxplots displaying the distribution of reconstructed VPD and SPEI values across drought severity categories. Left panelt: VPD-based drought classification (VPD-D, VPD-M, VPD-ND). Center: SPEI-based drought classification (SPEI-D, SPEI-M, SPEI-ND). Right panel: Combined drought clusters (COMB-D, COMB-M, COMB-ND) showing both, VPD (right axis) and SPEI (left axis) distributions. Boxes depict the interquartile range (IQR), with black dots representing outliers and black squares indicating the mean. Horizontal lines show medians. A box with stripes marks VPD.

Boxplots of each drought classification based on growing-season conditions (Fig. 7) confirmed a consistent separation of the climatic conditions across the categories. VPD-D years were characterized by significantly higher vapor pressure deficit values (mean VPD = 3.29 kPa), while SPEI-D years showed strongly negative soil moisture anomalies (mean SPEI = − 1.55). The COMB clusters showed similar distinctions, with COMB-D years averaging -1.53 for SPEI and 3.28 kPa for VPD, accompanied by notably reduced tree growth (mean RWI = 0.77) compared to COMB-ND years (mean RWI = 1.12) (Table 3). Notably, across all three drought classification frameworks (SPEI, VPD, and combined), approximately two-thirds of the years in the 200 years were categorized as drought years (severe or moderate, based on growing-season conditions), indicating a high baseline frequency of water stress conditions in this semi-arid region.

**Table 3:** Summary statistics of drought clusters.

Cluster type	Label	No. of Years	Mean SPEI	Mean VPD	Mean RWI
SPEI cluster	SPEI-D	32	− 1.552		0.760
	SPEI-M	97	− 0.443		1.000
	SPEI-ND	71	0.370		1.126
VPD cluster	VPD-D	43		3.291	0.921
	VPD-M	88		3.051	1.026
	VPD-ND	69		2.792	1.034
Combined	COMB-D	33	− 1.535	3.280	0.769
	COMB-M	96	− 0.438	3.054	1.01
	COMB-ND	71	0.369	2.834	1.124

Evaluation of drought severity showed a clear increase in the proportion of severe drought years (D), particularly in the VPD classification across the last two centuries. Severe atmospheric droughts comprised 46.4% of drought years in the most recent period (1971–2020), compared to 31.4% in 1821–1870 and 25.7% in 1921–1970. Similar trends, though less pronounced, were also observed in SPEI and combined classifications, COMB-D (from 18.8 to 34.5%) (Table S2).

The full chronology of individual drought years and their corresponding tree-ring width anomalies is provided in Supplementary Fig. S4. Despite the general agreement between individual and combined drought classifications, some discrepancies were observed. For instance, a number of years classified as VPD-D did not appear in the COMB-D cluster (Table 3 and Fig. S4), suggesting that atmospheric drought alone did not always result in combined drought conditions from a tree-growth perspective.

**Fig. 8:**
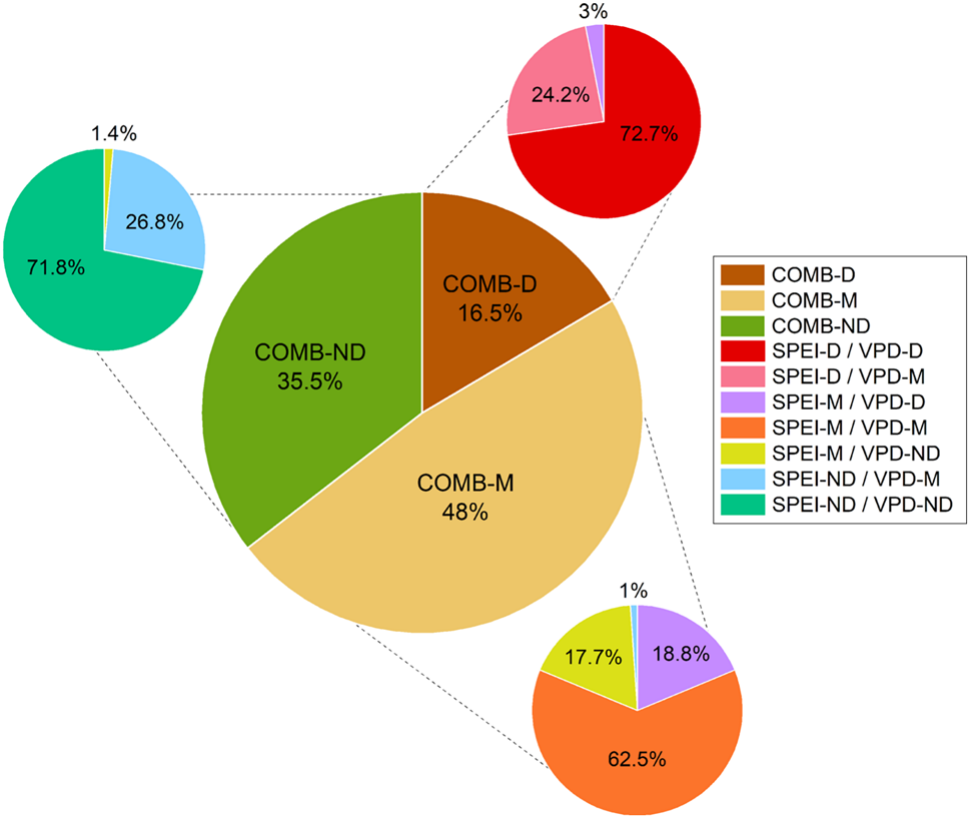
Composition of combined drought categories (COMB-D, COMB-M, COMB-ND) based on the combinations of VPD and SPEI classifications. The large central pie chart displays the relative frequency of each combined drought class across the full 200-year period. Smaller surrounding pie charts illustrate the composition of each COMB cluster by its constituent VPD/SPEI combinations.

**Fig. 9:**
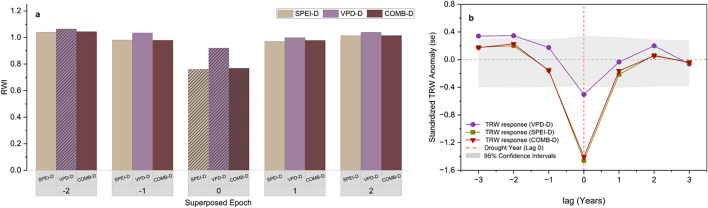
Tree growth responses to drought events classified by drought type. (**a**) Mean ring-width index (RWI) during a five-year superposed epoch window (lag –2 to + 2) for severe drought years identified as SPEI-dominated (SPEI-D), VPD-dominated (VPD-D), and combined droughts (COMB-D). Stripes indicate statistically significant anomalies at lag 0 (growing-season based drought year). (**b**) Standardized growth anomalies (SEA) showing growth departures relative to the 95% confidence interval (shaded area) derived from 1000 Monte Carlo simulations.

To further explore this pattern, we analyzed the compositional makeup of the combined drought clusters (Fig. 8). The results showed years classified as SPEI-D + VPD-M were more frequently grouped into the COMB-D cluster, while the reverse combination (SPEI-M + VPD-D) was often classified as COMB-M category, suggesting a stronger influence of soil moisture deficit on combined drought classification (Fig. 9).

### Tree growth responses to drought events

According to the Superposed Epoch Analysis (SEA), tree growth exhibited distinct responses to different types of droughts. The most substantial and persistent growth reductions occurred during SPEI-dominated drought years (SPEI-D), with a significant decline in the drought year (lag 0) of − 1.46 standardized units (*p* < 0.001), and a mean ring-width index (RWI) of 0.76. Compound drought years (COMB-D) showed similarly strong but slightly less severe reductions (− 1.41, *p* < 0.001, RWI = 0.77), while VPD-only drought years resulted in a more moderate anomaly of –0.50 (*p* < 0.001, RWI = 0.92).

Post-drought recovery was observed in all drought categories by lag + 1 and + 2, although growth remained below average (RWI < 1) for both SPEI-D and COMB-D years. In contrast, VPD-D years exhibited the mildest and shortest-lived growth reduction, with RWI values returning to above-average levels (RWI > 1) more quickly.

## Discussion

### Integrating multiple tree-ring parameters for an improved climate interpretation

Both proxies recorded signals of atmospheric and soil moisture deficits, but with distinct seasonal timing and response strength. δ^1^⁸O showed strong correlations with VPD and SPEI, revealing a peak correlation with VPD during the March–September season (r = 0.723), consistent with previous findings that oxygen isotopes in tree rings are strongly controlled by evaporative demand and atmospheric drought conditions during the growing season^35,36,72^, particularly in arid conditions^34,42^

The positive coefficient reflects the mechanistic relationship between higher δ^1^⁸O values in tree rings and increased evaporative demand on leaf water enrichment, linking δ^1^⁸O closely to atmospheric drought conditions^34,73^. This enhanced atmospheric signal may be further explained by two key mechanisms: the increasing vapor pressure deficit during the growing season, and the relatively low rate of oxygen exchange between xylem water (source water) and phloem sugars during cellulose synthesis. Together, these processes reduce the influence of source water and instead amplify the isotopic signature of leaf water, which is directly shaped by evaporative demand and atmospheric aridity^38,40^

Conversely, the observed negative correlation with SPEI**,** consistent with other studies^74^, suggests that during years with low soil moisture availability (i.e., years with negative SPEI values), evaporative demand was higher, resulting in stronger δ^1^⁸O enrichment. In addition to leaf-level evaporative enrichment, drought conditions may also lead to enriched δ^1^⁸O in soil water before uptake, particularly in shallow soils subject to intense evaporation. This secondary enrichment can further elevate δ^1^⁸O values in tree rings, especially in semi-arid environments^75^.

In contrast, TRW showed the highest sensitivity to soil moisture conditions during the early growing season, with a peak correlation with SPEI04-May (January–May; *r* = 0.65). This underscores the importance of cumulative soil water availability in the months leading up to and during the onset of radial growth, a pattern also reported in other tree-ring studies from semi-arid regions^76–78^. Juniper trees in the western Tien Shan exhibited the strongest growth sensitivity to SPEI^79^. The importance of soil moisture as a limiting factor for tree growth has been emphasized across a wide range of species, especially under current trends of increasing aridity and drying conditions^80,81^.

Overall, our findings suggest that δ^1^⁸O responds more strongly to mid- to late-season atmospheric drought, whereas antecedent soil moisture conditions exert a greater influence on TRW during the early growing season. These complementary seasonal sensitivities highlight the benefit of using both proxies to capture the full range of climate–growth interactions. Li et al.^82^ found that TRW and δ^1^⁸O in two conifer species with different rooting depths at the southern edge of the Tengger Desert were predominantly influenced by growing-season SPEI and summer relative humidity, respectively. It should be noted that longer accumulation periods may better reflect long-term hydrological drought persistence, but they showed weaker relationships with the proxy data in our study.

By applying a multiple regression framework combining δ^1^⁸O and TRW, we captured complementary aspects of drought variability and improved the representation of seasonal SPEI relative to the individual predictors. The improved model fit and validation statistics underscore the utility of dual-proxy approaches for enhancing climate signal detection, particularly in environments where univariate TRW–climate relationships are weak or non-stationary. Our findings support previous studies that have demonstrated the value of multi-parameter tree-ring approaches^83–86^.

A general limitation of proxy-based climate reconstructions is the assumption that the proxy–climate relationship remains sufficiently stable through time. In our case, the relatively short instrumental period does not allow moving-window analyses or split-period calibration/verification with longer subperiods. However, the identified relationships are consistent with known tree physiological mechanisms and were supported by LOOCV statistics, which increases confidence in the reconstruction. In addition, the use of coarse-resolution gridded climate data (0.5° CRU data) may introduce spatial mismatches in mountainous terrain; therefore, our reconstructions should be interpreted as reflecting regional hydroclimatic variability rather than exact site-specific conditions.

### Distinct influence of atmospheric and soil drought on tree growth

The diverging correlations between tree-ring parameters and growing-season drought indicators highlight distinct physiological responses of trees to soil versus atmospheric drought. The consistent negative correlation between δ^1^⁸O and VPD reflects increased evaporative enrichment of leaf water under high atmospheric demand, confirming that δ^1^⁸O is a reliable indicator of short-term vapor pressure deficit dynamics/ variations and reflects evaporative conditions in the study area. In contrast, TRW showed strong positive correlations with SPEI, indicating that tree growth is more closely tied to the temporal accumulation of soil moisture availability. Drier conditions reduce water availability for uptake and turgor maintenance, thereby limiting cambial activity and triggering growth decline.

Our drought classification and growth response analyses further support this differentiation. Years classified as severe drought conditions based on SPEI (SPEI-D), reflecting reduced growing-season moisture availability, produced the strongest and most prolonged growth reductions, nearly matching the declines observed in compound drought years (COMB-D). By contrast, years dominated by atmospheric drought alone (VPD-D) had milder and more transient effects, with faster recovery of growth. Additionally, the cluster composition analysis revealed that most COMB-D years were composed of SPEI-D + VPD-M combinations rather than VPD-D years. This asymmetry underscores that soil moisture deficits are the primary driver of compound drought years and of overall growth suppression in this semi-arid mountain ecosystem.

Stomatal closure is a key plant strategy to maintain hydraulic safety during drought^87^, but the degree and timing of closure are determined by the complex interplay between atmospheric demand and soil water availability. As VPD rises, stomata begin to close to limit water loss. However, under sustained high VPD and concurrent soil drying, stomatal closure becomes more complete, limiting gas exchange and further reducing cambial activity. Thus, although the initial response to atmospheric dryness may be modest, prolonged or combined stress from soil moisture deficits leads to sharper physiological constraints^88–90^.

Soil water availability and VPD are often tightly coupled, especially in semi-arid regions, where evapotranspiration is highly constrained by soil moisture^91,92^. In ecosystems with shallow soils, sparse vegetation, or high radiation loads—such as our open juniper forests in NE Iran—this coupling becomes even more pronounced. Root access to deeper soil layers, groundwater, or rock water can decouple this relationship and buffer drought impacts^93^, but *J. polycarpos* has a relatively shallow root system and relies primarily on superficial or stored water reserves^94^. Therefore, reductions in soil moisture can have a more immediate and pronounced impact on growth than rising VPD alone. In our study site, which receives low annual precipitation and features exposed soils with high evaporative losses, atmospheric dryness acts primarily to exacerbate soil moisture depletion^95^. This suggests that VPD acts as a more complementary or secondary role in growth limitation, consistent with recent findings showing strong soil moisture–VPD coupling under semi-arid conditions^90,96^.

SPEI integrates multi-month water deficits, capturing the cumulative nature of drought and the water budget required to sustain growth, whereas VPD reflects short-term atmospheric dryness and can vary substantially within a season^19,97^. While SPEI is widely used to represent hydroclimatic drought conditions related to soil moisture availability, it does not directly measure soil water content and should therefore be interpreted as an integrated climatic proxy rather than a direct soil moisture variable. Our findings are consistent with^98^, who also found that tree-ring δ^13^C in *J. polycarpos* was influenced by soil moisture availability, highlighting the species’ sensitivity to soil drought.

Altogether, these results suggest that soil drought is the dominant control on growth in *J. polycarpos* at our site. While VPD can amplify drought stress, especially in combination with low soil moisture, its standalone effect on growth is comparatively weaker. This underscores the importance of recognizing and managing compound drought risks, particularly in arid mountain ecosystems where growth sensitivity to soil moisture dominates^99,100^. Atmospheric drought plays a critical but reinforcing role and should not be viewed in isolation^89,101,102^.

### Implications for long-term forest resilience and climate change

Understanding how forest ecosystems respond to drought is essential for anticipating climate impacts and guiding forest management. In the arid and semi-arid mountain regions of West Asia, our findings confirm that long-term deficits in soil moisture, as captured by SPEI, leave more persistent and severe imprints on tree growth than short-term fluctuations in atmospheric demand (VPD). While atmospheric drought can intensify stress when combined with depleted soil water, it is rarely the sole driver of major growth suppression.

In all three classification schemes (SPEI, VPD, and combined), approximately two-thirds (64–66%) of years were categorized as drought years (years with a dry growing season). This high prevalence of drought years underscores the persistent pressure of both soil and atmospheric moisture deficits on forest productivity and resilience. These results align with emerging evidence from other drought-prone ecosystems^103,104^, where increasing drought frequency and severity threaten long-term forest productivity and recovery^99,103–106^. Although the total number of drought years has remained relatively stable over time, we observed a clear increase in the proportion of severe drought years in the most recent period (1971–2020). This was particularly evident for VPD, where severe events rose from 31.4% (1821–1870) to 46.4% (1971–2020), and for combined droughts (COMB-D), which increased from 18.8 to 34.5% in the same timeframe. Similar increases are also seen in SPEI-D proportions. These results align with broader regional and global trends showing increased drought intensity, even in the absence of a rise in drought frequency^21,43,107^. Based on tree-ring-based reconstructions of June–August VPD, 43) also found an increasing trend in summer VPD during recent centuries in Central Europe and the Mediterranean region over the past four centuries, supporting the signal of intensifying atmospheric drought across the World. This emerging drought intensification trend, driven by both soil and atmospheric aridity, poses particular risks to the resilience of semi-arid mountain forests such as those in northeastern Iran^2,3^. Species growing in these regions may face growing limitations on (biomass) productivity, regeneration, and long-term persistence.

To sustain ecosystem functioning under future drought scenarios, adaptive management strategies must prioritize soil moisture retention through measures that enhance infiltration, reduce surface runoff, and minimize evaporative losses. In parallel, identifying or selecting drought-resilient species capable of tolerating both edaphic and atmospheric stress will be key to climate-smart reforestation efforts.

## Conclusions

Our study highlights the value of integrating multiple tree-ring parameters —tree-ring width (TRW) and stable oxygen isotopes (δ^1^⁸O) —to disentangle the impacts of soil and atmospheric drought on tree growth in a semi-arid mountain ecosystem. While TRW primarily captured the influence of cumulative soil moisture during the early growing season, δ^1^⁸O proved highly sensitive to mid-to-late season atmospheric dryness, particularly vapor pressure deficit (VPD). Together, these proxies offered a seasonally complementary view of tree-climate interactions.

Our drought classification framework highlights the differentiated roles of soil and atmospheric drought in shaping tree growth trajectories. While soil moisture availability emerged as the primary constraint on growth in this semi-arid mountain ecosystem, atmospheric dryness—particularly when co-occurring with soil drought—acted as a compounding stressor. These insights demonstrate the importance of disentangling distinct drought mechanisms to understand tree vulnerability and resilience under changing climatic conditions.

Crucially, our analysis of long-term drought classifications across four 50-year periods (1821–2020) revealed a notable shift toward more intense droughts in recent decades. This emerging trend of drought intensification, even in the absence of more frequent drought events, underscores the growing stress on mountain forests under a warming climate.

These findings underscore the vulnerability of semi-arid mountain ecosystems in West Asia to intensifying climate stress, particularly from compound drought events. Effective management strategies must therefore prioritize soil moisture retention and the selection of drought-resilient species that can tolerate both atmospheric and edaphic moisture limitations. Long-term, multi-proxy reconstructions such as those presented here provide critical insights into the historical dynamics of drought impacts and offer valuable guidance for anticipating and mitigating future risks under climate change.

## Supplementary Information

Supplementary Information.

## Data Availability

The datasets generated during and/or analysed during the current study are available from the corresponding author on reasonable request.
