# Supplementary material for: Tree-ring width and δ18O-derived hydroclimatic reconstructions allow a distinction between soil and atmospheric drought in the Mountain Forests of Northeastern Iran
Source: Sci Rep. 2026 May 19;16:15601. doi: 10.1038/s41598-026-52364-3 (PMC13187265; doi:10.1038/s41598-026-52364-3)
Supplement: Supplementary file 1 — Supplementary Information. [file 41598_2026_52364_MOESM1_ESM.docx]

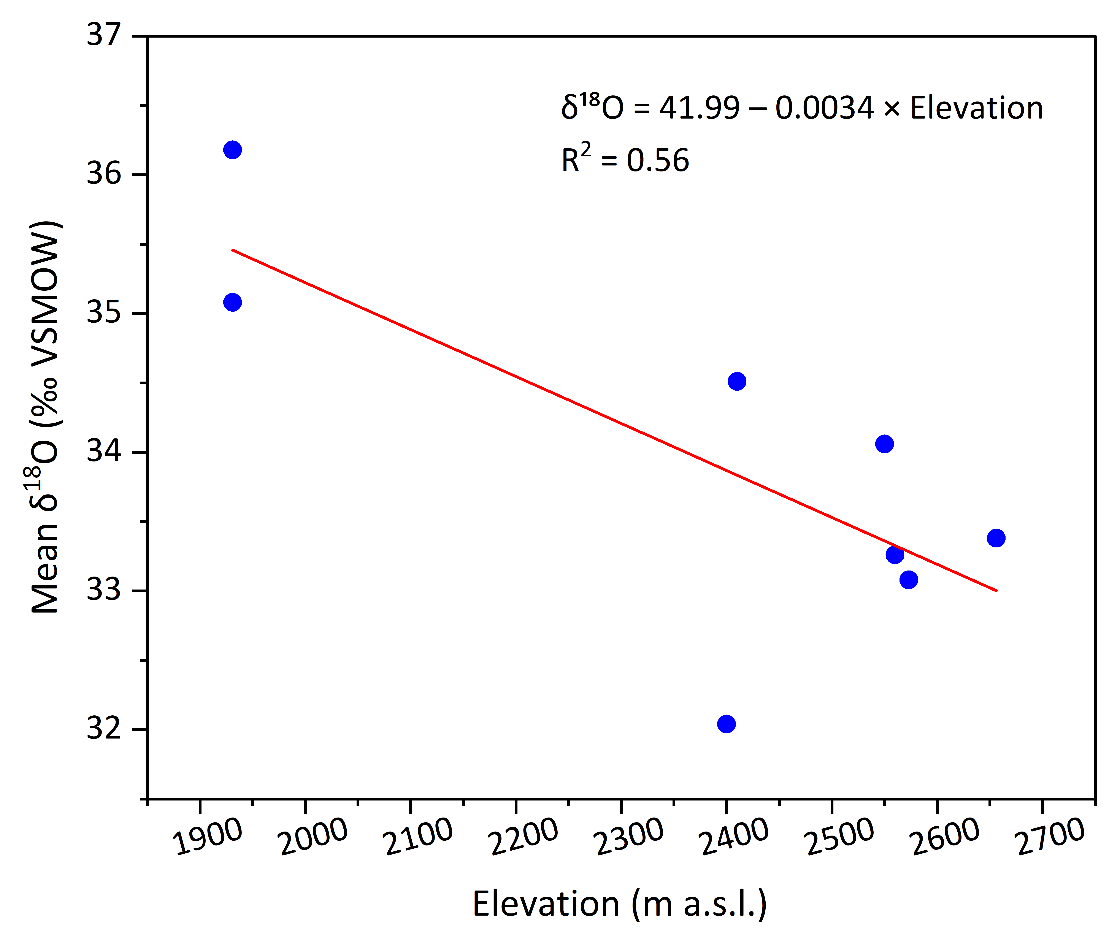


Figure S1. Linear regression between tree sampling elevation and mean δ¹⁸O values across individual trees within the study site. The analysis revealed a statistically significant negative relationship (r = –0.75, p < 0.01), indicating a decrease of 0.3386‰ per 100 m of elevation gain. The model explained 56.4% of the variance in δ¹⁸O values (R² = 0.56), reflecting a moderate but consistent altitudinal effect on the isotopic composition of tree-ring cellulose.


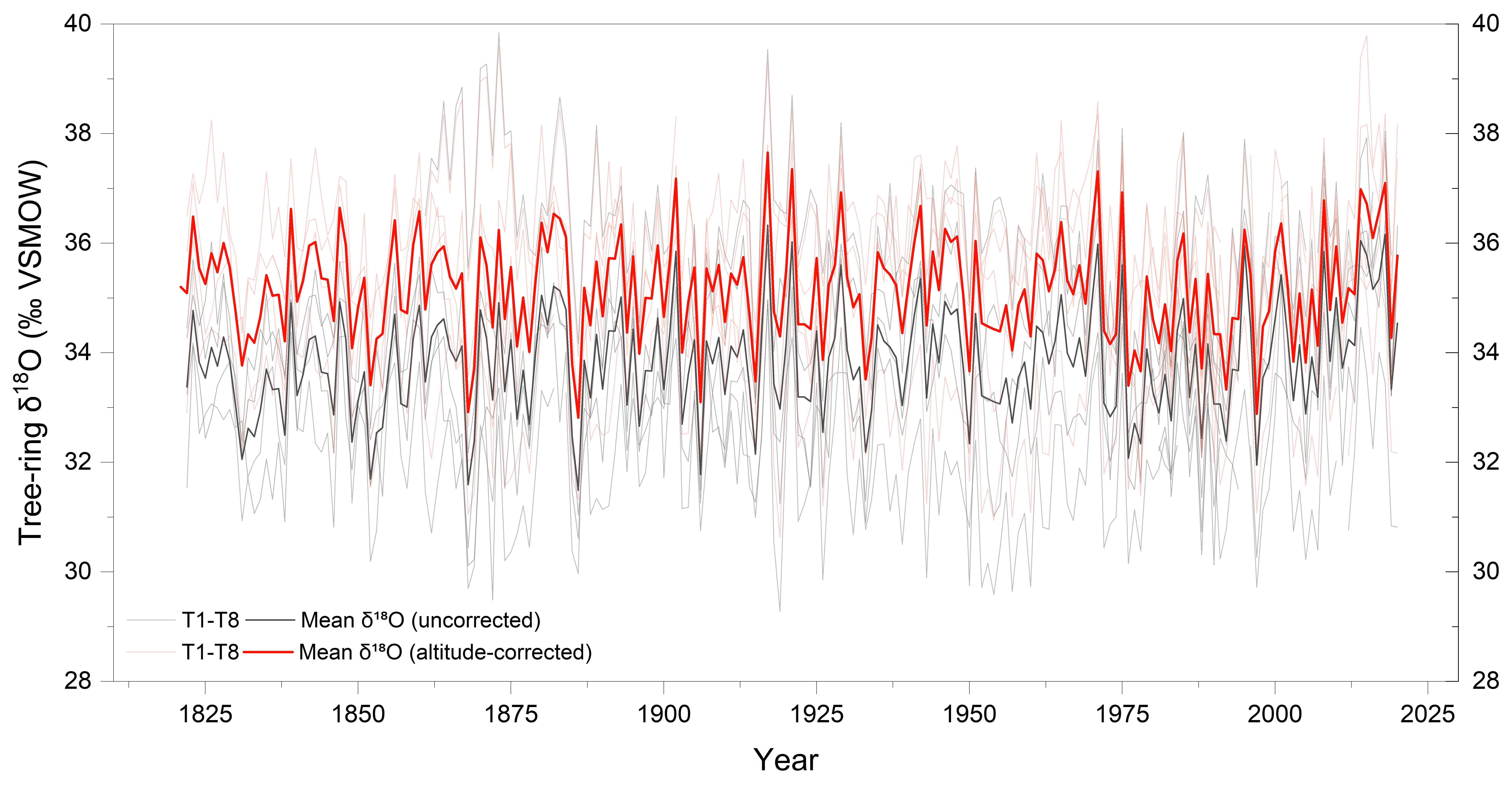


Figure S2. Comparison of tree-ring δ¹⁸O time series before and after altitude correction. Thin grey and red lines represent individual uncorrected and corrected δ¹⁸O series from all sampled trees, respectively, illustrating the strong temporal coherence among series. The black line shows the mean uncorrected δ¹⁸O chronology, while the red line represents the mean chronology after normalization to a reference elevation of 2000 m a.s.l. The altitude correction reduces systematic differences in mean δ¹⁸O values among trees associated with elevation, while preserving the year-to-year variability and common climatic signal.

**
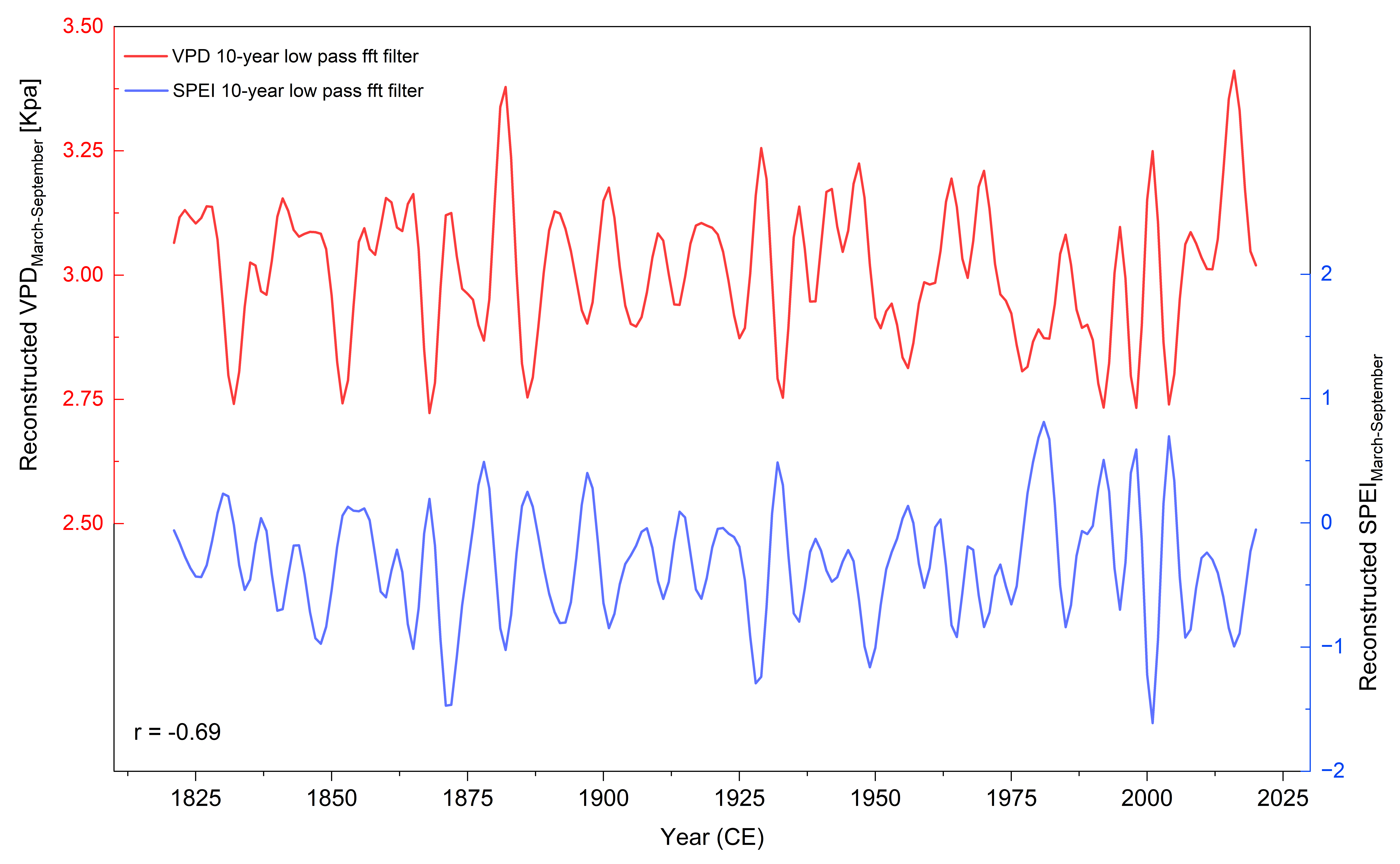
**

Figure S3. Reconstructed growing-season (March–September) atmospheric drought (VPD; red line, left axis) and hydroclimatic drought conditions related to soil moisture availability (SPEI; blue line, right axis) over the period 1821–2020, shown as 10-year low-pass FFT–filtered time series. The VPD reconstruction is based on the δ¹⁸O chronology of J. polycarpos, whereas the SPEI reconstruction is derived from a combined δ¹⁸O and tree-ring width (TRW) multi-proxy model. The two reconstructions exhibit coherent decadal-scale variability, with a Pearson correlation of r = -0.69

**
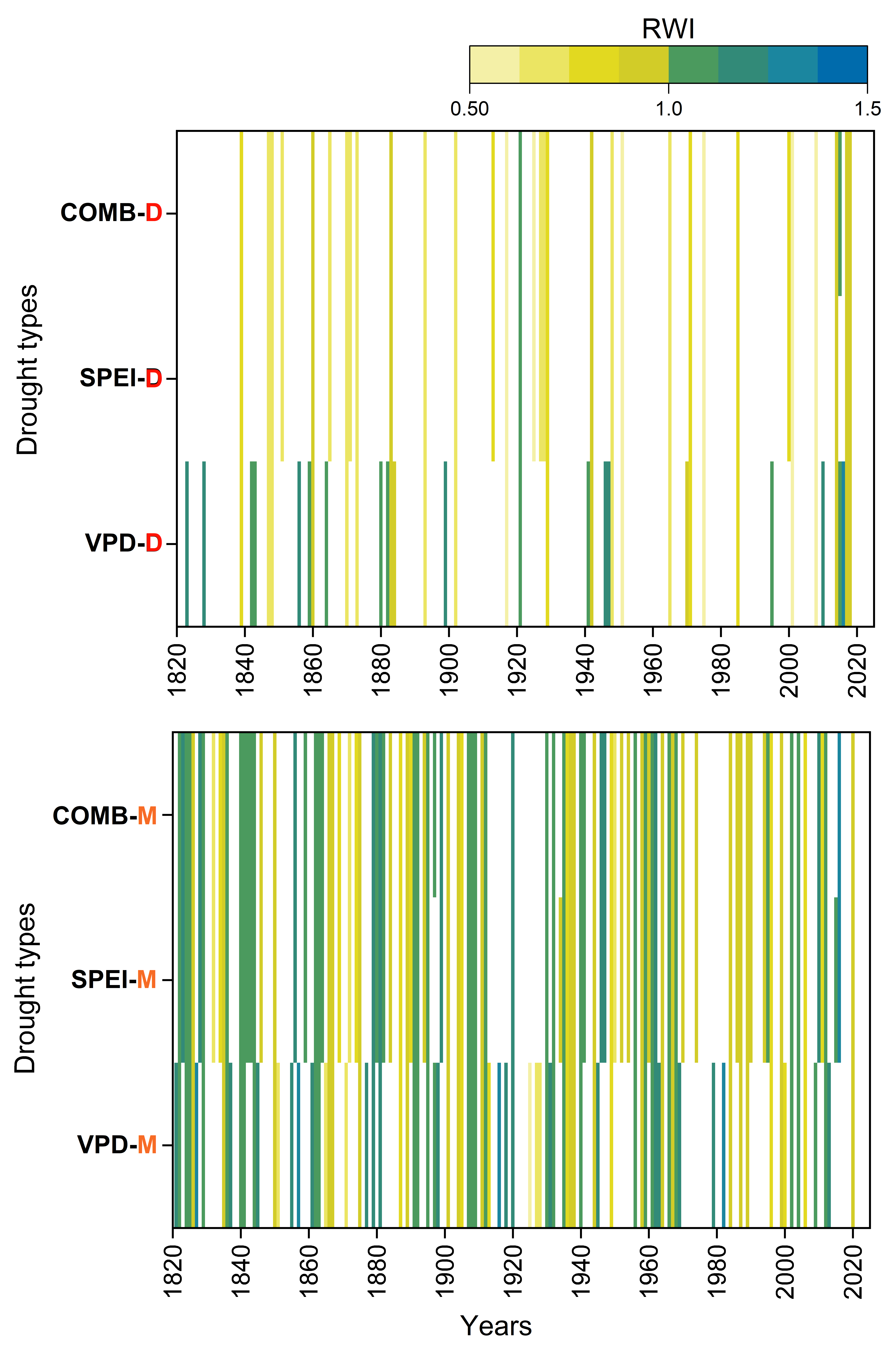
**

Figure S4. Tree-ring width index (RWI) during years classified as severe (D) and moderate (M) droughts based on individual and combined drought types. SPEI refers to hydroclimatic drought conditions related to soil moisture availability, VPD represents atmospheric drought (vapor pressure deficit), and COMB denotes combined drought conditions derived from both SPEI and VPD classifications using cluster analysis. Each vertical bar represents one year, colored according to the mean tree growth. The upper panel shows RWI during SPEI-D, VPD-D, and COMB-D years, while the lower panel depicts the same for moderate drought (M) classifications.

*
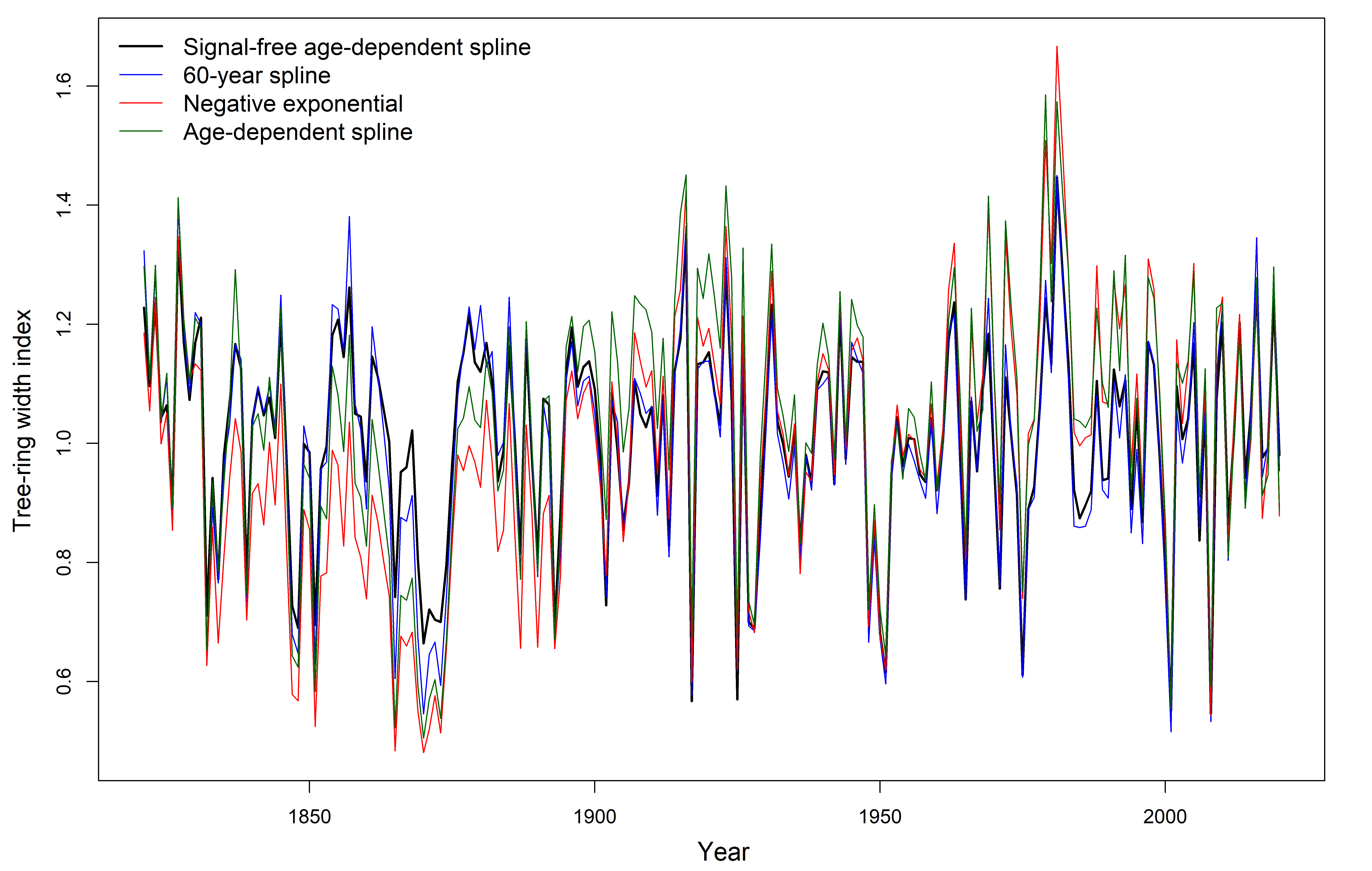
*

Figure S5. Comparison of tree-ring width index (RWI) chronologies derived using different detrending methods (signal-free age-dependent spline, 60-year spline, negative exponential, and age-dependent spline). All chronologies exhibit highly similar interannual variability, demonstrating that the main growth signal is robust to the choice of detrending method.

Table S1. Frequency of drought classifications in 50-year intervals based on SPEI-only, VPD-only, and combined (SPEI + VPD) K-means clustering. Drought years are categorized as D (Drought), M (Moderate), and ND (Non-Drought).

| Period | SPEI-D | SPEI-M | SPEI-ND | SPEI-D / (D+M) |
| --- | --- | --- | --- | --- |
| 1821–1870 | 6 | 26 | 17 | 6 / 32 = 18.8% |
| 1871–1920 | 8 | 24 | 18 | 8 / 32 = 25.0% |
| 1921–1970 | 9 | 27 | 14 | 9 / 36 = 25.0% |
| 1971–2020 | 9 | 20 | 22 | 9 / 29 = 31.0% |
| Period | **VPD-D** | **VPD-M** | **VPD-ND** | **VPD-D / (D+M)** |
| 1821–1870 | 11 | 24 | 14 | 11 / 35 = 31.4% |
| 1871–1920 | 10 | 23 | 17 | 10 / 33 = 30.3% |
| 1921–1970 | 9 | 26 | 15 | 9 / 35 = 25.7% |
| 1971–2020 | 13 | 15 | 23 | 13 / 28 = 46.4% |
| Period | **COMB-D** | **COMB-M** | **COMB-ND** | **COMB-D / (D+M)** |
| 1821–1870 | 6 | 26 | 17 | 6 / 32 = 18.8% |
| 1871–1920 | 8 | 25 | 17 | 8 / 33 = 24.2% |
| 1921–1970 | 9 | 26 | 15 | 9 / 35 = 25.7% |
| 1971–2020 | 10 | 19 | 22 | 10 / 29 = 34.5% |

Table S2. Summary of the strongest correlations between tree-ring proxies (δ¹⁸O, TRW) and SPEI calculated over different time windows and months during the calibration period (1984–2020). Each SPEI variant integrates the climatic water balance over a given number of preceding months (e.g., SPEI05 = 5-month cumulative water balance). The table highlights the time periods and SPEI versions yielding the strongest climate–proxy relationships.

| Proxy | SPEI variant | Category | Best Time Window | Highest correlation |
| --- | --- | --- | --- | --- |
| δ¹⁸O | SPEI01 | Strongest short-term correlation | March-July | -0.683 |
|  | SPEI05  (5-mo SPEI) | **Strongest observed correlation** | September | -0.653 |
|  | SPEI07 | Selected growing-season reconstruction target (Mar–Sept cumulative) | September | -0.617 |
| TRW | SPEI01 | Strongest short-term correlation | February-May | 0.630 |
|  | SPEI04 | **Strongest observed correlation** | May | 0.652 |
|  | SPEI07  (7-mo SPEI) | Selected growing-season reconstruction target(Mar–Sept cumulative) | September | 0.565 |
| TRW + δ¹⁸O | SPEI07 | Final reconstruction model | September | 0.715 |
